# Perspective: Vegan Diets for Older Adults? A Perspective On the Potential Impact On Muscle Mass and Strength

**DOI:** 10.1093/advances/nmac009

**Published:** 2022-02-02

**Authors:** Jacintha Domić, Pol Grootswagers, Luc J C van Loon, Lisette C P G M de Groot

**Affiliations:** Division of Human Nutrition and Health, Wageningen University, Wageningen, The Netherlands; Division of Human Nutrition and Health, Wageningen University, Wageningen, The Netherlands; Department of Human Biology, School of Nutrition and Translational Research in Metabolism (NUTRIM), Maastricht University Medical Centre+, Maastricht, The Netherlands; Institute of Sports and Exercise Studies, HAN University of Applied Sciences, Nijmegen, The Netherlands; Division of Human Nutrition and Health, Wageningen University, Wageningen, The Netherlands

**Keywords:** plant-based food, animal-based food, plant-based diet, aging, protein, sustainable food

## Abstract

Consumers are increasingly encouraged to consume more plant-based foods and lower their consumption of foods from animal origin. Concurrently, older adults are recommended to consume an adequate amount of high-quality dietary protein for the prevention of age-related muscle loss. In the current Perspective article, we discuss why it may not be preferred to consume a vegan diet at an older age. Our perspective is based on the proposed lower bioavailability and functionality of proteins in a vegan diet due to the matrix of the whole-food protein sources, the lower essential amino acid (EAA) content, and specific EAA deficiencies in proteins derived from plant-based foods. We propose that a vegan diet increases the risk of an inadequate protein intake at an older age and that current strategies to improve the anabolic properties of plant-based foods are not feasible for many older adults. We provide recommendations for further research to substantiate the remaining knowledge gaps regarding the consequences of a vegan diet on skeletal muscle mass and strength at an older age.

## Teaser

**Statement of Significance**: To our knowledge, this is the first article that provides an evidence-based perspective regarding the potential impact of a vegan diet on muscle mass and strength in older adults. The current perspective is highly relevant considering the envisaged shifts towards sustainable dietary guidelines.

## Introduction

Several countries are adapting to dietary guidelines that recommend increasing the consumption of plant-based foods and thereby lowering the consumption of animal-based foods (1, 2). This transition towards plant-based eating is primarily driven by environmental factors and is endorsed by the EAT-Lancet commission and the European Union's call for Europe-wide sustainable dietary guidelines before the end of 2030 (3, 4).

Plant-based diets can be classified according to the relative contribution of animal- and plant-based foods within the diet. A vegan diet is considered the strictest plant-based diet and excludes all foods from animal origin (5). The increased consumption of fruits and vegetables that concur with a vegan diet may improve the dietary intake of important nutrients and bioactive compounds. In accordance, vegan diets have been associated with potential health benefits, such as improving cardiovascular risk factors (6–9). These indications are promising for several subgroups of the adult population. However, the consequences of consuming a vegan diet at an older age on skeletal muscle mass and strength remain unknown. Concurrently, the loss of muscle mass and strength within the aging population is an emerging public health concern (10).

The age-related loss of muscle mass and strength becomes apparent approximately in the fifth decade of life and may result in sarcopenia. Sarcopenia is a muscle disorder that is characterized by low muscle strength in combination with low muscle mass or quality (11). In community-dwelling older adults, the estimated prevalence of sarcopenia ranges from 9.9% to 40.4% depending on the definition used (12). Sarcopenia increases the risk for frailty, falls, hospitalization, impaired recovery, and mortality (11, 13). As such, in view of the envisaged transition towards more plant-based dietary guidelines, it is urgently needed to underscore the potential impact of a vegan diet on muscle mass and strength at an older age.

The aim of the current Perspective article is to discuss the potential consequences of a vegan diet on muscle mass and strength in adults aged 65 y and older. Thereby, we extend our evaluation from data on isolated plant-based protein sources towards the impact of consuming whole meals and diets. This whole-diet, or so-called holistic, approach has gained more emphasis in the recent years as it considers all food component interactions within meal matrices (14, 15). Burd and colleagues (15) recently highlighted the importance of such an approach when assessing the effects of dietary interventions, focusing on meals and diets rather than single food sources, since food components interact differently when consumed as part of a meal rather than in isolation.

The current article *1*) provides a short overview of the current status of knowledge regarding dietary protein recommendations for older adults, *2*) discusses the quality and functionality of both isolated plant-based proteins and proteins within a vegan meal, *3*) highlights why current strategies to improve the anabolic properties of a vegan diet may not be feasible for older adults, *4*) explains that a vegan diet increases the risk of an inadequate protein intake in older adults, *5*) provides an overview of the existing evidence regarding vegan diets and the potential impact on muscle mass and strength in older individuals, and *6*) provides suggestions for future research regarding this aspect.

## Current Dietary Protein Recommendations for Older Adults

An effective strategy to attenuate the rate of decline in muscle mass and strength with advancing age is ample physical activity combined with sufficient intake of high-quality dietary protein (16, 17). Dietary protein contains indispensable nutrients called essential amino acids (EAAs) that are vital for maintenance of muscle mass and strength throughout life. The consumption of dietary protein induces hyperaminoacidemia, which stimulates muscle protein synthesis (MPS) and inhibits muscle protein breakdown via various pathways. The balance between MPS and muscle protein breakdown (i.e., muscle protein turnover) determines muscle maintenance, loss, or growth. When MPS exceeds breakdown consistently, this leads to the accretion of newly synthesized muscle proteins and eventually muscle growth (18, 19).

The ability of a protein source to alter muscle protein balance is dependent on its digestion and absorption, splanchnic amino acid uptake, plasma amino acid availability, the transportation of amino acids to the muscle, the subsequent uptake of amino acids by the muscle, and intramuscular signalling (20). Furthermore, the EAA content of a protein source is pivotal for the postprandial stimulation of MPS (21–25). When a protein source lacks 1 EAA, all other amino acids appear to be oxidized rather than used for MPS (26). It is suggested that EAAs themselves may function as nutritional signals with the potential to directly stimulate MPS and inhibit protein breakdown. For example, the EAA leucine is shown to have the ability to stimulate MPS by activating the mammalian target of rapamycin complex 1 (mTORC1) pathway, and subsequently activating mRNA translation (22, 27, 28).

Older adults are recommended to consume sufficient high-quality protein to maintain muscle mass and physical function and to support healthy aging (13, 16, 17, 29). The RDA for dietary protein is 0.8 g/kg body weight per day [g/(kg·d)] for all adults aged 18 y and older (30). However, the current RDA is primarily obtained from nitrogen balance studies in young adults and may not be representative for older individuals (13, 31). Older adults have been observed to exhibit lower sensitivity to protein consumption, known as age-related anabolic resistance. As such, older adults may require a higher dose of protein intake to adequately stimulate MPS (13). Expert groups emphasize that a daily dietary protein intake of 0.8 g/(kg⋅d) is too low for the maintenance of muscle mass in individuals aged ≥65 y, and have repeatedly proposed to increase the RDA for older adults to approximately 1.0–1.2 g/(kg⋅d), with specific attention to timing and quality of the consumed protein (13, 16, 17, 29).

## The Protein Quality of a Vegan Diet

The quality of a protein source (i.e., a whole-food product or protein in its isolated form) relates to its ability to meet metabolic demands and to support growth and maintenance of body protein mass (32). The quality of a protein source is determined by its EAA content, the digestibility of the protein, and subsequent bioavailability of the protein-derived amino acids. The bioavailability of an amino acid is the proportion of the consumed amino acid that is fully digested and absorbed (31, 33–35). Together, all these factors contribute to the functionality of the protein—that is, the ability of the amino acids within the protein to serve their purpose after absorption, for example, to synthesize new proteins in the body or to serve their role in the regulation of neurotransmission or fluid balance.

The recommended method to evaluate protein quality is amino acid scoring. Until recently, the Protein Digestibility Corrected Amino Acid Score (PDCAAS) was the recommended means to express the quality of a protein source. The PDCAAS was calculated based on a single value of fecal crude protein digestibility. This factor was criticized and considered as a major shortcoming and led to the adoption of a new amino acid scoring method, the Digestible Indispensable Amino Acid Score (DIAAS). The DIAAS differs from the PDCAAS by using true ileal amino acid digestibility for each individual EAA instead of a single fecal crude protein digestibility value for evaluating the quality of a protein source, since this reflects the bioavailability of the EAAs more accurately (31). A short explanation of the DIAAS can be found in **Text Box 1**.

Text Box 1 **The DIAAS**

The DIAAS is currently the recommended method by the FAO to evaluate the protein quality of a food, meal, or diet. The DIAAS is based on the EAA composition of the food source and the ileal digestibility of each EAA. The DIAAS of a protein source is determined by its most limiting digested EAA. As the DIAAS of a protein source increases, the quality of that protein source increases. The DIAAS classification is as follows: <75, no quality claim; 75–99, high-quality protein; ≥100, excellent-quality protein. A DIAAS ≥100 indicates that the protein source has no limiting EAA. These foods may be used as complementary sources to foods with a lower DIAAS to achieve better protein quality in a diet (31).

The DIAAS is acknowledged as the best method currently available to assess protein quality, but still exhibits several limitations that are essential to consider when evaluating the protein quality of a vegan diet (34, 36). One of these limitations is that the true ileal digestibility of the EAA does not always accurately reflect bioavailability when it comes to dietary protein sources that have undergone a certain degree of processing (e.g., at the manufacturer or heat treatment while cooking at home). This is mainly the issue for the amino acids lysine, methionine, cysteine, threonine, and tryptophan (34, 37, 38). According to Batterham (38), the ratio of retained lysine to the ileal digestible lysine in pigs was only 0.36 after consuming a cottonseed meal. The ratios were higher after consuming a meat and a soybean meal (0.60 and 0.75). For methionine, these values were 0.38 for the cottonseed meal and 0.45 for both the meat and the soybean meal. It was concluded that heat processing affects the availability, but not the digestibility of these amino acids, and as such, that the true ileal digestibility of these amino acids overestimates their availability, especially in meals that exhibit a lower protein quality. Another limitation is that the DIAAS does not provide insight on how the absorbed amino acids stimulate downstream physiological targets (i.e., its functionality), such as the stimulation of MPS (39). Furthermore, the DIAAS values of many foods that are consumed within a vegan diet, such as meat analogues, nuts, and seeds, are not yet available.

To date, most research into DIAAS exclusively focused on raw and isolated food products and proteins (36, 39, 40). Protein concentrates (≥25% protein) and isolates (≥90% protein) are derived by the removal of nonprotein constituents. Taking it a step further, proteins can be processed via enzymatic hydrolyzation to provide protein hydrolysates (41). Subsequently, any effects of other food components on the bioavailability of the protein-derived amino acids are excluded. However, proteins and food products are generally consumed as part of a meal rather than in their isolated form and it is essential to consider the interplay between food components within such a meal that may affect amino acid bioavailability and subsequently their functionality (32, 42, 43). Taking these factors into account, in the following paragraphs we separately discuss that *1*) the protein quality of most isolated plant-based foods and proteins is inferior to those of animal origin and *2*) the quality of vegan mixed meals and diets is uncertain and that calculating its DIAAS based on blending isolated food sources does not account for the food component interaction(s) within a single meal or within a specific diet.

### Isolated plant-based foods and proteins exhibit inferior protein quality compared with animal-based foods and proteins

**FIGURE 1:**
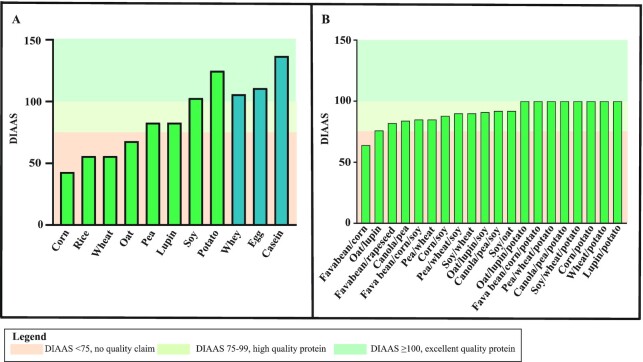
An overview of the DIAAS as determined using the adult reference values for several plant- and animal-based food products (A) and plant-based food blends (B). Data derived from Herreman et al. (40) (supplementary data). DIAAS, Digestible Indispensable Amino Acid Score.

Protein quality and amino acid content vary widely among animal- and plant-based foods and proteins. The amino acid profile of most animal-based foods and proteins can be considered complete, meaning that these foods and proteins do not exhibit a limiting amino acid, and their DIAAS often reflects excellent protein quality (**Figure 1**A). On the other hand, the plant-based foods and proteins from which data regarding protein digestibility are currently available are frequently limiting in 1 or more EAA, resulting in lower DIAAS, and show a high heterogeneity regarding their amino acid profiles (40, 44–48). Gorissen et al. (44) observed that plant-based protein isolates exhibit, on average, an 11% lower EAA content than animal-based protein isolates, with a wide variety in EAA profiles between the different plant-based protein isolates. Specifically, the EAA contents of oat, lupin, wheat, and hemp exhibit an EAA content below the amino acid requirements as established by the WHO (49) and their EAA contents display 21%, 21%, 22%, 23%, and 23% of total protein, respectively. On the other hand, soy, brown rice, pea, corn, and potato do meet the amino acid requirements established by the WHO and display 27%, 28%, 30%, 32%, and 37% of EAAs from total protein. Plant-based foods and proteins show particularly high heterogeneity regarding methionine and lysine contents. Interestingly, plant-based foods that are high in methionine often appear to be low in lysine and vice versa (44). An incomplete EAA profile results in an increased oxidation of the other EAAs. Consequently, these EAAs will not become available for MPS (26).

Another notable difference in EAA content between isolated animal-based foods and proteins and those of plant origin is that isolated plant-based foods and proteins generally exhibit a lower proportion of the anabolic EAA leucine (44). The leucine content of the plant-based protein isolates observed by Gorissen et al. (44) varied from 5.1% in hemp to 13.5% in corn, with an average leucine content of 7.1% ± 0.8%. The animal-based protein isolates in that study exhibited a higher average leucine content of 8.8% ± 0.7%. Zaromskyte et al. (50) recently found that in older adults specifically, the leucine content of isolated proteins appears to play an important role in the postprandial regulation of MPS. It should be noted, however, that in the context of mixed meals, the regulatory role of leucine on MPS remains uncertain. It has been observed that whole eggs (containing 18 g protein and 17 g fat) elicit a greater MPS response when compared with egg whites (18 g protein, 0 g fat) in young individuals, despite a similar plasma leucine response (51). Although the mechanism behind this finding remains unclear, other components within the food matrix of a mixed meal, as discussed in more detail below, may also modulate the MPS response rather than aminoacidemia alone. An important notion on this is that this observation was made in young individuals and the regulatory role of leucine in MPS appears to be less evident at a younger age (50). As such, the specific role of leucine within the context of mixed meals in older individuals requires further research.

Herreman et al. (40) combined information from different datasets to calculate the DIAAS of 17 foods and a number of food blends. Several of these DIAAS values are presented in Figure 1. All but 1 of the animal-based foods assessed in their study contained protein of excellent quality (DIAAS ≥100). With respect to the plant-based foods, the DIAAS values as calculated with the adult reference values ranged from 43 to 125, with only soy and potato showing excellent protein quality. Pea and lupin both showed a DIAAS of 83, indicating high protein quality. The remaining plant-based foods were not classified as high or excellent protein quality as a result of their low DIAAS values (40). Another study calculated that the DIAAS values for seitan, tofu, soya milk, and pea emulsion containing pea protein isolate were, respectively, 28, 97, 117, and 60, again emphasizing the high heterogeneity in protein quality between plant-based foods (47).

The difference in amino acid digestibility between isolated animal- and plant-based protein was recently illustrated in healthy young males (52). In that study, Pinckaers and colleagues (52) observed significantly greater increases in plasma EAA, leucine, lysine and methionine concentrations after the ingestion of 30 g of milk protein concentrate (MPC) compared with a similar amount wheat protein hydrolysate. A smaller, but still significant, increase in these plasma EAA values was observed after the consumption of MPC as compared with a blend containing both MPC and wheat protein hydrolysate, illustrating improvements in EAA availability with increasing quality of the protein source. Similar findings were observed in older men (53).

The differences in EAA availability observed by Pinckaers and colleagues were, however, not reflected in the effects on MPS. The wheat protein hydrolysate led to sufficient availability of EAAs to stimulate MPS in these young men (52). However, in older individuals, isolated protein of low quality has been observed to be inferior in stimulating MPS when compared with higher quality protein (53, 54). A recent meta-analysis (54) found that protein of high quality, which was based on total amino acid profiles, leucine content, and DIAAS, elicited a greater increase in postprandial and resistance exercise–induced MPS of 0.012 (0.01–0.02) and 0.014 (0.01–0.02) than did dose-matched, low-quality proteins in older individuals. These values related to a mean difference of 41% and 33% between protein of high quality and the control group for postprandial and resistance training–induced MPS. The effect on MPS appeared to be more evident at a more advanced age. Nonetheless, there was no effect of protein quality on lean body mass and strength in older adults. This discrepancy may be explained by several factors. As discussed in detail by the authors as well, the acute measurement of MPS is predominantly performed under strictly controlled circumstances and may not directly relate to longer-term outcomes that ensue under free-living conditions, especially without the assessment of muscle protein breakdown. Furthermore, with the exception of 1 study, all studies used a DXA scan to assess lean body mass. Since lean body mass assessed via a DXA scan also includes other soft tissues in addition to muscle mass, small effects on muscle mass may have been overlooked. The small sample of studies available for meta-analysis regarding these outcomes and inconsistent assessment methods between studies may have contributed to these discrepant findings as well (54). It should be noted that the extent to which older adults respond to protein consumption is highly dependent on their physical activity level and the amount of protein consumed (53, 55, 56).

### Combining isolated plant-based foods is only a crude estimation of the protein quality of a vegan meal

The heterogeneity in amino acid profiles of plant-based foods allows for making combinations of 2 or more complementary sources to achieve higher DIAAS values (Figure 1B) (33, 40, 44). When different plant-based foods are combined properly within a meal, metabolic amino acid demands can be met by smaller portions. This would lead to a meal with an improved nutritional efficiency. In particular, plant-based foods that are of high protein quality on their own, such as potato and soy, have the potential to complement the plant-based foods of lower protein quality. For example, a food blend of pea, wheat, and potato leads to a DIAAS of 100, whereas their individual DIAAS values are 83, 56, and 125 (40). Since foods are mostly consumed as part of a meal rather than in isolation, this provides an opportunity to adequately combine different plant sources within a meal to obtain a higher protein quality.

Unfortunately, this strategy provides merely a rough estimation of the actual DIAAS of a meal. The so-called food matrix and the processing or preparing of a meal affect amino acid bioavailability and cannot be disregarded. For example, antinutritional factors (ANFs) are components in foods that interfere with the availability of nutrients. ANFs formed during food processing, naturally occurring in the food product, or formed during genetic modification of crops, such as Maillard compounds, oxidized sulfur amino acid, d-amino acids, and trypsin inhibitors, limit amino acid bioavailability (31, 32, 42, 43). Dietary trypsin inhibitors, mainly found in soybeans and other grain legumes, have been shown to reduce protein digestibility up to 50% in animal studies (42). Furthermore, tannins and phytates, mainly present in cereals and grain legumes appear to reduce protein digestibility by as much as 23% and 10%, respectively (42). Alternatively, 2 studies observed that, with regard to animal-based foods, other components within the food matrix may positively modulate the muscle anabolic response as well (51, 57). In addition to the study addressed earlier that observed an increased MPS response following the consumption of whole eggs versus egg whites (51), Elliot et al. (57) observed that the ratio of threonine uptake relative to the amount of threonine ingested, which represented the net MPS response, was 312% and 91% greater for whole milk than for fat-free milk (*P* < 0.05) and isocaloric fat-free milk in young individuals. However, this aspect is scarcely studied and it remains to be established how the food matrix affects the anabolic properties of a vegan meal in older adults.

Most food components are taken into account in the determination of the DIAAS of a single food product, since they directly affect protein digestibility (31). This was illustrated in a recent study that assessed the true ileal digestibility of both soy-based tofu and soya milk in pigs and observed lower true ileal digestibility values of the EAAs methionine and cysteine in tofu when compared with soya milk, resulting in a substantially lower DIAAS in tofu (47). Additionally, in piglets, the apparent ileal nitrogen digestibility of raw pea diets has been observed to be 69.1% and 69.5%, whereas this was 83.7% and 85.4% for the diets based on pea protein isolates (58).

Although the DIAAS of isolated foods provides valuable information, the nutrients and ANFs within the food matrix interact differently when consumed as part of a meal (59). The procedure for the calculation of DIAAS for mixed meals and diets as proposed by the FAO does not take this interaction between different food sources into account. The calculation is based on the summation of DIAAS values of the isolated foods rather than using the ileal digestibility of the mixed meal or diet (31). It would indeed not be feasible to assess the ileal digestibility of all the countless possibilities of different food combinations; nonetheless, it is pivotal to consider that any food component interaction within the meal and diet is not reflected in these calculations. This makes it complex to draw conclusions regarding the protein quality of a vegan meal or diet, especially when considering that plant-based foods generally exhibit more ANFs than animal-based foods (43).

### Increasing portion sizes to improve the anabolic properties of plant-based meals is not a feasible strategy for older adults

Increasing portion sizes has been proposed as a strategy to increase the intake of protein and EAAs and subsequently compensate for the lower anabolic properties of plant-based foods and proteins (33, 46). In line with this, Gorissen et al. (53) observed that ingesting 60 g of wheat protein hydrolysate substantially increased MPS in healthy older adults, whereas 35 g did not. Similarly, Yang et al. (56) showed that rates of MPS increased after the consumption of 40 g of soy protein isolate but did not significantly increase when a lower dose of 20 g was consumed (56). In accordance, the Dutch Ministry of Health recommends a 1.3 times higher protein intake for all individuals ≥18 y following a vegan diet due to the suggested lower digestibility of protein from plant-based foods (60). This would lead to a protein recommendation of 1.3–1.5 g/(kg ⋅ d) for vegan older adults, considering the already proposed increased dietary protein recommendations.

In view of the well-defined anorexia of aging (61), increasing portion sizes to compensate for the proposed lower anabolic properties of vegan meals will be challenging for most older adults (53, 56). The anorexia of aging relates to the reduction in appetite that arises in the course of aging. A meta-analysis observed that older adults, on average, experience a significantly greater fullness of 37% ± 73% after an overnight fast compared with younger individuals. Average postprandial hunger and hunger after an overnight fast appeared to be 39% ± 30% and 25% ± 24% lower, respectively, in older adults compared with younger individuals (*P* < 0.05 for both outcomes) (62). The prevalence of the anorexia of aging ranges between 15% and 30% in community-dwelling older adults and is observed to be higher in institutionalized (31%) and hospitalized (31.5%) older adults (63). Anorexia of aging has been associated with multiple comorbidities and an increased mortality risk (63). The underlying pathophysiology is highly complex and not fully understood, but higher circulating concentrations of anorectic hormones in older adults have been suggested to play a role (63, 64).

Considering the above, the potential satiating effect of vegan meals should not be overlooked. This has been assessed in several studies (65–69). Kristensen et al. (65) observed that a high-protein, legume-based meal [beans and peas; 3.5 MJ, 28% of energy (en%) fat, 19 en% protein, 53 en% carbohydrates, 25 g fiber/100 g] led to lower ratings of appetite, hunger, and prospective food intake, and higher ratings of satiation and a reduced ad libitum energy intake when compared with an isocaloric high-protein, meat-based meal (pork meat; 28 en% fat, 19 en% protein, 53 en% carbohydrate, 6 g fiber/100 g). The low-protein, legume-based meal (28 en% fat, 9 en% protein, 62 en% carbohydrate, 10 g fiber/100 g) showed a similar satiation and palatability as the high-protein, meat-based meal. Similar findings were observed after the consumption of a mycoprotein-based meal in comparison to an isonitrogenous meal containing chicken (66, 67). On the other hand, other studies did not observe a higher satiating effect of a vegan meal in comparison to an omnivorous meal (68, 69). These mixed findings may result from the different foods included in the test meals, the different study populations, or differences in fiber content between test meals. Fiber content varied between and within the studies. The studies that did not observe a difference in satiety between test meals displayed smaller differences in fiber content of the test meals (1.0 g vs. 5.0 g per meal; 1.0 g vs. 4.4 g per meal) (68, 69) than the studies that did (25 g/100 g vs. 6 g/100 g; 10.1 g vs. 16.8 g per meal; 11 g vs. 3 g per meal) (65–67). It should be noted that these studies were carried out in young individuals and, to date, no studies have assessed the effects of adopting a vegan meal or diet in older adults. Additional research concerning this aspect in older adults would provide essential information regarding the satiating effects of vegan meals and diets in this population. This would add valuable insights to the presently posed question of whether vegan meals or diets can provide an adequate amount of available EAAs for older adults. If substantial increases in portion sizes are indeed not feasible, strategic menu planning with the help of a nutritionist or dietitian, while also considering the unknown consequences of the food matrix on protein functionality, will be essential to optimize the amount of protein and available EAAs provided by a vegan meal.

## A vegan diet increases the risk of an inadequate protein intake in older adults

**FIGURE 2:**
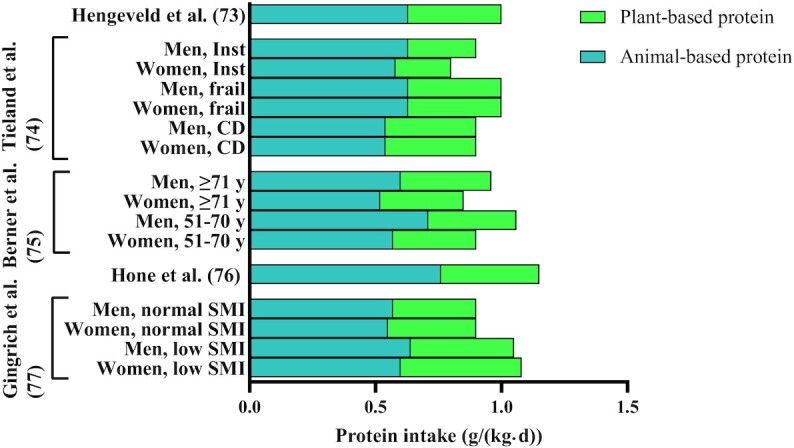
Animal- and plant-protein intake in older adults aged ≥65 y from Western countries. Data derived from references 73–77. CD, community-dwelling; Inst, institutionalized; SMI, skeletal muscle index.

Animal-based foods currently form the major source of dietary protein in older adults living in Western societies (70–75). As shown in **Figure 2**, studies have repeatedly observed a high relative contribution (≥60%) of animal-based foods to protein intake in older adults (73–77). Daily dietary protein intake in all but one of these studies was below, or near, 1.0 g/(kg ⋅ d), which is at the lower end of the proposed increased recommendations for dietary protein intake (13, 16, 17, 29). Additionally, a recent meta-analysis on 4 cohorts and 4 national surveys (78) reported that approximately one-fifth of older adults had a daily dietary protein intake below 0.8 g/(kg ⋅ d). Nearly half of the older adults had a daily protein intake below 1.0 g/(kg ⋅ d).

The main contributors to dietary protein intake in the older population are meat, dairy, and cereal products (73–75). Since animal-based food products provide such a high contribution to dietary protein intake in the current diet of older adults, exchanging omnivorous portions with vegan portions of the same size may strongly affect the amount of food and the source of protein ingested. The lower protein density of plant-based foods poses the risk that, in such a scenario, daily dietary protein intake will decrease even further if vegan alternatives are not carefully considered. Taking into account the lower protein quality of most plant-based foods as well, this may be problematic, considering that a substantial proportion of the older population does not meet the proposed increased recommended intake for dietary protein (73–75, 78).

Therefore, when aiming to eliminate the intake of animal-based foods, the animal-based foods would have to be carefully replaced by a sufficient amount and wide variety of plant-based foods. A study that modelled dietary intake data in adults aged >51 y assessed the potential impact of *1*) doubling plant-based food products as habitually consumed or *2*) doubling high-protein, plant-based food products in their diet (legumes, nuts, seeds, and soy) on protein intake (79). According to these models, doubling the intake of plant-based foods as habitually consumed, and thereby reducing the intake of animal-based foods, would reduce protein intake per ideal body weight by 22%. On the other hand, doubling the consumption of high protein, plant-based foods did not affect habitual protein intake. This highlights the importance of careful consideration when animal-based products are fully replaced by plant-based products, with particular attention to both protein content and quality.

Although more research is needed, the above suggests that switching to a vegan diet would increase the risk of not meeting the recommended dietary intake for protein intake in older individuals even further, especially in those with a decreased appetite. Data regarding dietary consumption of older adults following a vegan diet are lacking, making it complex to speculate which plant-based foods should replace the foods of animal origin if an older individual would switch to a vegan diet. It was recently observed that vegan adults aged 20 y and older consumed over twice the amounts of high-protein alternatives (i.e., legumes, vegetarian meat alternatives, nuts, soy, and plant milk) compared with adults who did consume meat (80). Still, these vegan adults showed a low relative intake of high protein sources (contributing to one-fifth of their total energy) compared with vegetarians (one-fourth) and meat eaters (one-third). As such, animal-based food consumption was not fully replaced by high protein, plant-based alternatives but rather by an increased consumption of a variety of plant-based foods (80). Another study in young adults observed that vegans had a significantly lower consumption of dietary protein compared with omnivores (13.1 en% vs. 19.1 en%) (81). Nevertheless, these observations were made in young individuals and may differ from the actual dietary intake of an older individual. Future studies that assess dietary consumption in vegan older adults would provide valuable insights into their dietary patterns and their effects on protein intake. We acknowledge that we are still at the beginning of the protein transition, and the number of vegan older adults may not be high presently (82, 83). Nevertheless, as numbers of individuals consuming plant-based diets will grow as a result of the envisaged shifts in dietary guidelines, such insights will become indispensable in the near future.

## The potential impact of vegan diets on muscle mass and strength at an older age

So far, we have presented data indicating that a vegan diet can lead to decreases in overall protein intake and in the intake of high-quality protein sources in older adults, which may have consequences for muscle health. Here, we focus on the evidence that investigated the potential direct effects of plant-based diets on muscle mass and strength in this population.

### Intervention studies

To date, only 2 intervention studies have investigated the consequences of vegan meals and diets on muscle mass and strength in older adults (**Table 1**). Monteyne et al. (84) observed no significant differences in MPS rates between a 3-d mycoprotein-based vegan diet compared with an omnivorous diet in either exercised or nonexercised leg muscle. Both diets, however, provided a high protein intake of as much as 1.8 g/(kg ⋅ d). As discussed in detail earlier, the differences in anabolic properties between animal- and plant-based protein sources may diminish with higher doses of protein (33, 46, 53, 56). The feasibility of such a diet for older adults, however, remains debatable, as discussed in detail earlier. As yet, to the best of our knowledge, studies assessing the effects of a vegan diet with a protein content that is representative and feasible for the older population are lacking.

**TABLE 1:** Overview of intervention studies that investigated the effects of a vegan or a vegetarian meal or diet on muscle-related outcomes in older adults^1^

								Favors^2^		
Study	*n*	Age	Design	Duration	Intervention	Control	Outcome	V	O	N
Vegan										
Monteyne et al. (84)	19	66 ± 1	RCT	3 d	Mycoprotein-based vegan diet	Omnivorous diet	MPS			X
					Protein content: 1.8 ± 0.00 g/(kg·d)	Protein content: 1.8 ± 0.00 g/(kg·d)				
Kim et al. (85)	12	65 ± 2	Crossover RCT	9 h	Egg-based breakfast	Cereal-based breakfast	WB protein synthesis			X
					Protein content: 26.0 g	Protein content: 25.5 g	WB protein breakdown		X	
							WB net protein balance		X	
							MPS			X
Vegetarian										
Campbell et al. (88)	19	58 ± 2	Parallel-group	13 wk	Self-selected lacto-ovo-vegetarian diet	Habitual omnivorous diets	Fat-free mass		X	
					Protein content: 0.78 ± 0.1 g/(kg·d)	Protein content: 1.0 ± 0.08 g/(kg·d)	Whole-body muscle mass		X	
					Resistance exercise	Resistance exercise	Type II muscle fiber area			X
							Muscle strength (1RM)			X
							Muscle metabolites	X		
Haub et al. (87)	21	65 ± 5	RCT	12 wk	Partially self-selected lacto-ovo-vegetarian diet	Partially self-selected beef-containing diet	Fat-free mass			X
					Protein content: 1.15 ± 0.1 g/(kg·d)	Protein content: 1.03 ± 0.3 g/(kg·d)	CSA Vastus lateralis muscle			X
					Resistance exercise	Resistance exercise	Muscle strength (1RM)			X
							Muscle metabolites			X
Pannemans et al. (86)	12	69 ± 4	Crossover	2 wk per diet	Diet A: 5.3 en% AP, 5.0 en% VP		Nitrogen balance		X	
					Diet B: 14.5 en% AP, 5.1 en% VP		WB protein flux		X	
					Diet C: 5.0 en% AP, 15.1 en% VP		WB protein oxidation		X	
							WB protein synthesis		X	
							WB protein breakdown		X	
							WB net protein synthesis	X		

1 AP, animal protein; CSA, cross-sectional area; en%, percentage of energy; MPS, muscle protein synthesis; N, neutral; O, omnivorous meal or diet; RCT, randomized controlled trial; V, vegan or vegetarian meal or diet; VP, vegetable protein; WB, whole body; 1RM, 1 repetition maximum.

2 The −X− represents whether the outcome of the related study favored the vegan diet, the omnivorous diet or none (neutral outcome).

Furthermore, 1 study investigated the difference in acute anabolic response between a cereal-based breakfast and an egg-based breakfast followed by a standardized lunch in older adults (85). The egg-based breakfast resulted in a significantly greater whole-body net protein balance than the cereal-based meal as a result of a greater suppression of whole-body protein breakdown, which is consistent with the findings of Pannemans et al. (86) discussed below. These effects, however, diminished after the consumption of a standardized lunch meal. There was no meal effect on both whole-body protein synthesis and MPS, despite the greater EAA content and the greater plasma EAA and leucine response following the egg-based breakfast.

Due to the limited amount of evidence available, we discuss intervention studies that focused on predominantly plant-based diets in older adults (i.e., vegetarian diets) as well (86–88) (Table 1). A notion on this is that vegan and vegetarian diets may exhibit very different nutrient profiles and should not be conflated. Assessing the effects of vegetarian diets may provide us with some information regarding the anabolic effects of lowering animal-based food consumption. However, these findings should be interpreted with caution since vegan diets may induce different anabolic properties since they display a different nutrient profile. Nevertheless, with the limited amount of evidence currently available regarding vegan diets in older adults, these studies cannot be omitted from the current discussion.

Pannemans and colleagues (86) observed that a diet consisting of primarily vegetable protein sources led to a nonsignificant lesser inhibition of whole-body protein breakdown compared with a diet consisting primarily of animal protein sources in older women, resulting in a significantly less positive whole-body net protein balance (86). Interestingly, postabsorptive protein breakdown was suppressed by 40% after the older women switched from the low-protein diet to the high-animal-protein diet, whereas this suppression was only 27% when they switched to the high-vegetable-protein diet (86). Another study observed no differences in muscle mass and strength between a lacto-ovo-vegetarian diet and a beef-containing diet in older men while following a 12-wk resistance training program (87), whereas Campbell et al. (88) observed that consuming a lacto-ovo-vegetarian diet for 12 wk with concurrent resistance exercise training resulted in declines in fat-free mass and whole-body muscle mass as opposed to increases observed when consuming an omnivorous diet. Protein quantity in the diets differed profoundly between, and within, these intervention studies, which may explain these discrepant findings (86–88).

### Observational data

Several observational studies assessed associations between plant-based food intake and muscle-related outcomes. These observational studies showed mixed results (**Tables 2** and **3**) (89–101). Hengeveld et al. (101) observed that 10-g lower vegetable protein intake was associated with a 20% increase in “pre-frailty or frailty” incidence in nonfrail individuals, whereas others observed that higher intake of animal, not vegetable, protein [OR, quartile 4 vs. quartile 1 (reference) and 95% CI of animal protein intake: 0.48; 0.26–0.87) decreased frailty incidence in Spanish older adults (97). However, the substantially lower total protein (67 g/d vs. 92 g/d) and higher relative plant-protein (40% vs. 33%) intake as observed by Hengeveld et al. (101) may provide an explanation for these contradictory findings. Similarly, the higher relative plant-protein intake (53%) observed by Coelho-Junior et al. (91) likely contributed to the discrepancy between their findings and those of other studies (28% and 33%) (95, 97).

**TABLE 2:** Overview of observational studies that assessed the associations between plant- and animal-protein intake and physical outcomes measures in adults aged >60 y^1^

						Associations	
Study	*n*	Men	Age, y	Design	Outcome	P	A
Chan et al. (96)	2726	52%	≥65	4-y prospective cohort	6-m walking speed	—	—
					Narrow walk speed	—	—
					Average step length	—	—
McLean et al. (95)	646	46%	60–85	6-y prospective cohort	Annual loss of handgrip strength	—	√
Sandoval-Insausti et al. (97)	1822	49%	>60	3.5-y prospective cohort	Frailty incidence	—	√
Hengeveld et al. (101)	2154	49%	70–81	4-y prospective cohort	Frailty incidence	—	—
					Pre-frailty or frailty incidence	√	—
Coelho-Junior et al. (91)	90	13%	60–85	Cross-sectional	Fast walking speed	√	X
					Usual walking speed	—	—
					Handgrip strength	—	—

1 A, animal-based protein intake; P, plant-based protein intake; √, the protein is favorably and significantly associated with the outcome in the fully adjusted model; X, the protein is negatively and significantly associated with the outcome in the fully adjusted model; –, the protein did not show a significant association with the outcome in the fully adjusted model.

**TABLE 3:** Overview of observational studies that assessed the associations between plant- and animal-protein intake and muscle mass in adults aged >60 y^1^

						Associations	
Study	*n*	Men	Age, y	Design	Outcome	P	A
Chan et al. (96)	2726	52%	≥65	4-y prospective cohort	Appendicular skeletal muscle mass	√	—
McLean et al. (95)	646	46%	60–85	6-y prospective cohort	Baseline arm lean mass	—	—
Houston et al. (89)	2066	47%	70–79	3-y prospective cohort	Lean mass	—	√
					Appendicular lean mass	—	√
Verreijen et al. (92)	1561	48%	70–79	6-y prospective cohort	CSA Vastus lateralis muscle	—	—
Isanejad et al. (93)^2^	554	0%	65–71	3-y prospective cohort	Lean mass		
					Baseline	—	√
					3-y follow-up	—	√
					Trunk lean mass		
					Baseline	—	√
					3-y follow-up	—	—
					Appendicular lean mass		
					Baseline	—	—
					3-y follow-up	√	√
Miki et al. 2017 (94)	168	58%	≥65	Cross-sectional	SMI	√	√
Huang et al. (90)	327	Unknown	65–85	Cross-sectional	Muscle mass	√	NA
					SMI	√	NA
Yaegashi et al. (98)	277	42%	≥65	Cross-sectional	Muscle mass	—	√^3^
					Appendicular muscle mass	—	√^3^
					Appendicular SMI	—	—
Alexandrov et al. (100)	76 633	41%	65–75	Cross-sectional	Creatinine excretion	—	√^4^

1 A, animal-based protein intake; CSA, cross-sectional area; NA, not assessed; P, plant-based protein intake; SMI, skeletal muscle mass index; √, the protein is favorably and significantly associated with the outcome in the fully adjusted model; –, the protein did not show a significant association with the outcome in the fully adjusted model.

2 Data were derived from a 3-y intervention study assessing the effects of calcium and vitamin D supplementation in older adults. The current table shows results from the total population in that study.

3 Only in female participants aged ≥75 y.

4 Not in men aged >75 y.

With regard to muscle mass, animal-based protein consumption showed more positive associations than plant-based protein (Table 3) (89, 93, 98, 100). A 3-y prospective cohort study observed significant associations with animal protein intake and lean mass (β = 6.58 ± 2.75; *P* = 0.02) and appendicular lean mass (β = 4.13 ± 1.52; *P* = 0.006) (89). These findings are in accordance with another study that assessed these associations within a broader age range (29–86 y) (102). That study observed that participants who were within the highest quartile of total protein intake, relating to a median total protein intake of 101 g/d for men and 93 g/d for women, had a greater leg lean mass (men: 17.6 kg vs. 17.1 kg, *P* = 0.005; women: 11.7 kg vs. 11.4 kg, *P* = 0.06) than those in the lowest quartile, which related to a median total protein intake of 64.9 g/d for men and 57.8 g/d for women. A similar trend was observed across the different quartiles of animal-protein intake (leg lean mass in men: 17.1 kg vs. 17.6 kg, *P* = 0.002; women: 11.5 vs. 11.7 *P* = 0.003), but not plant-protein intake (102). As discussed in detail previously, the high quality of animal protein and its effects on muscle mass has been well established (46, 53, 56, 103, 104). As such, it is not surprising that this is reflected in the observational data.

The favorable associations between animal-protein intake and muscle mass were most profound in women (89, 93, 94, 98, 100). Two other studies observed inferiority of predominantly plant-based diets on muscle mass in older women as well (105, 106). Nieman et al. (106) observed that vegetarian older women tended to have a lower muscle mass than other women within their age group. In that study, vegetarian older women exhibited a midupper arm muscle area of 33 cm^2^, whereas this was 38.2 cm^2^ in nonvegetarians, albeit not significantly different from the vegetarians. In accordance, another study observed that a vegetarian diet was associated with a lower muscle mass index than an omnivorous diet in healthy middle-aged women (105). On the other hand, others also observed plant-protein intake to be positively associated with muscle mass in their fully adjusted models (90, 93, 94, 96). Chan et al. (96) observed a significant trend across the quartiles of vegetable protein intake and appendicular skeletal muscle mass. Participants in the highest quartile of vegetable protein intake [vegetable-protein intake >0.93 g/(kg ⋅ d)] showed a significantly smaller reduction in appendicular lean mass than those in the lowest quartile [vegetable-protein intake ≤ 0.44 g/(kg ⋅ d); 0.270 kg vs. 0.349 kg].

Whether the associations resulted from a direct causal relation between plant-protein intake and muscle mass remains to be established. Higher intakes of plant protein have been related to improved diet quality (107). This is also illustrated by the significant positive association between vegetable-protein intake and quadriceps strength that diminished after correction for vegetable and fruit intake in the study of Sahni et al. (102). In addition, a prospective cohort study observed that, when compared with an overall diet of good quality, poor- and medium-quality diets, as based on the Healthy Eating Index, were associated with substantially higher incidence of frailty (101). This emphasizes the need for more intervention studies to assess the direct consequences of a vegan diet for muscle mass and strength in older adults as well as to explore the effect of diet quality on these outcomes.

## Conclusions and Perspectives

On account of the high ecological impact of animal-based food production, we are in the midst of a transition towards the consumption of more plant-based diets, and the demand for sustainable dietary guidelines is growing. Although there are many important health benefits of vegan diets for several subgroups of the adult population with regard to consuming more fruits and vegetables, the lesser anabolic properties of plant-based foods may compromise muscle mass and strength at an older age. Based on this, we feel that it may not be preferred for older adults to consume a vegan diet.

Observational data indicate that the majority of older adults do not consume the recommended intake for dietary protein. Adequate consumption of high-quality dietary protein combined with ample physical activity is pivotal for the prevention of sarcopenia. A vegan diet increases the risk of an inadequate protein intake, since plant-based foods generally exhibit a lower protein density and suboptimal EAA content. Furthermore, isolated plant-based foods and proteins display an inferior protein quality compared with animal-based foods and proteins, which is reflected in their anabolic potential. The substantial increase in portion sizes that is needed to consume an adequate amount of protein and EAAs from a strict vegan diet and to overcome the inferior anabolic properties of plant-based foods is not feasible for many older adults, especially for those with a decreased appetite and a low physical activity level.

There remain, however, major gaps in the literature. First, evidence regarding protein consumption in vegan older adults is lacking, emphasizing the need for research into the dietary patterns of vegan older adults with specific attention to protein consumption. Second, the satiating effects of vegan meals and diets in older individuals are unknown. Exploring these effects would provide valuable insights into how a vegan diet would affect dietary (protein) intake in this population. Third, while making smart food combinations provides the opportunity to compile a vegan meal that has an improved nutritional efficiency, it remains to be established how such a meal affects EAA bioavailability and protein functionality. Last, to date, evidence regarding the direct consequences of a vegan diet on muscle-related outcomes in older adults is limited exclusively to 2 intervention studies.

Essentially, whether a vegan diet can adequately promote muscle mass and strength can only be established within well-designed intervention studies. Future studies are needed to assess the impact of a vegan diet provided by whole foods and/or supplemented by isolated protein supplements on muscle-related outcomes in older adults. We emphasize the importance of exploring both the acute effects on MPS as well as the longer-term effects on, for example, muscle mass, strength, and physical functioning, since the acute effects on MPS do not directly reflect longer-term changes in muscle health. Such studies should consider portion sizes that are feasible for older adults. Furthermore, since we are addressing a wide-ranging population, future studies should target different subgroups within the older population to assess whether the consequences of adhering to a vegan diet may differ between these subgroups.

Until the potential negative consequences of a vegan diet on muscle-related outcomes later in life are ruled out, we infer that it may not be preferred to consume a vegan diet for adults aged 65 y and older. When the elimination of animal-based food consumption is still deemed necessary—for example, due to cultural or personal preferences—careful consideration should be given to the plant-based foods that replace the animal-based foods in the diet of an older individual. In such a scenario, increasing protein intake by supplementation of isolated plant-based proteins or EAAs in adequate quantities may be a strategy to decrease the risk of an inadequate protein and EAA intake.

## Notes

Supported by a grant from the Regio Deal Foodvalley (162135).

LJCvL has received research grants, consulting fees, speaking honoraria, or a combination of these for research on the impact of exercise and nutrition on muscle metabolism, which include funding from companies that produce dairy, meat, and/or plant-derived proteins. A full overview on research funding is provided at: https://www.maastrichtuniversity.nl/l.vanloon. LCPGMdG and PG have received grants for research on the impact of nutrition (and exercise) on muscle metabolism, from the Dutch government (ZonMw), the European Union (H2020), and Top Institute Food and Nutrition, which pre-competitively includes funding from food industry (small and medium sized enterprises and larger enterprises). LCPGMdG is an editor on *Advances in Nutrition* but played no role in the Journal's evaluation of the manuscript.

Author disclosures: The authors report no conflict of interest.

Perspective articles allow authors to take a position on a topic of current major importance or controversy in the field of nutrition. As such, these articles could include statements based on author opinions or point of view. Opinions expressed in Perspective articles are those of the author and are not attributable to the funder(s) or the sponsor(s) or the publisher, Editor, or Editorial Board of *Advances in Nutrition*. Individuals with different positions on the topic of a Perspective are invited to submit their comments in the form of a Perspectives article or in a Letter to the Editor.

Abbreviations used: ANF, antinutritional factor; DIAAS, Digestible Indispensable Amino Acid Score; EAA, essential amino acid; en%, % of energy; MPC, milk protein concentrate; MPS, muscle protein synthesis; PDCAAS, Protein Digestibility Corrected Amino Acid Score.
